# Quality of life of ice hockey players after retirement due to concussions

**DOI:** 10.2217/cnc-2020-0007

**Published:** 2020-08-04

**Authors:** Anna Gard, Niklas Lehto, Åsa Engström, Pashtun Shahim, Henrik Zetterberg, Kaj Blennow, Niklas Marklund, Yelverton Tegner

**Affiliations:** 1 Department of Clinical Sciences Lund, Neurosurgery, Lund University, Skåne University Hospital, Lund 222 41, Sweden; 2 Department of Applied Physics, Luleå University of Technology, Luleå 971 87, Sweden; 3 Department of Health Sciences, Luleå University of Technology, Luleå 971 87, Sweden; 4 Clinical Neurochemistry Laboratory, Institute of Neuroscience & Physiology, Sahlgrenska Academy at University of Gothenburg, Sahlgrenska University Hospital, Mölndal 431 41, Sweden

**Keywords:** ice hockey, IES-R, postconcussive syndrome, quality of life, SF-36, sports-related concussion

## Abstract

**Background:**

Sports-related concussion (SRC) is increasingly recognized as a potential health problem in ice hockey. Quality of life (QoL) in players retiring due to SRC has not been thoroughly addressed.

**Materials & methods:**

QoL using the Sports Concussion Assessment Tool 5th Edition, Impact of Event Scale-Revised and Short Form Health Survey was measured in Swedish ice hockey players who retired due to persistence of postconcussion symptoms or fear of attaining additional SRC.

**Results:**

A total of 76 players were assessed, on average of 5 years after their most recent SRC. Overall, retired players had a high burden of postconcussion symptoms and reduced QoL.

**Conclusion:**

Retired concussed ice hockey players have a reduced QoL, particularly those retiring due to postconcussion symptoms. Symptom burden should be continuously evaluated and guide the decision to retire.

## Lay abstract

Concussions sustained while playing ice hockey are increasingly recognized as a potential health problem. Long-term consequences for players retiring due to concussions have not been fully investigated. Therefore, we evaluated symptoms, quality of life and post-traumatic stress in former Swedish ice hockey players who retired due to postconcussion symptoms or to a fear of attaining additional concussions. We found that retired ice hockey players with a history of concussions had a low quality of life and high post-traumatic stress, particularly in players with a high symptom burden. We recommend that symptom burden should be continuously evaluated and guide the decision to retire.

The speed of modern ice hockey, where forceful body-to-body contact is common, puts the athlete at risk for high-energy impacts. Overall, the injury incidence is high [1–8]. The head is the most commonly injured body part and concussion ranks among the most frequent injuries [8]. In the 1980s, concussion incidence was approximately 20 concussions/1000 game hours in elite ice hockey [9] and several recent reports show an increased incidence [9–12]. To date, the number of reported concussions in Swedish ice hockey has also increased and is estimated at approximately 150 concussions/1000 game hours [9].

A sports-related concussion (SRC) is defined as a mild traumatic brain injury (mTBI) induced by biomechanical forces during sports game or practice [13]. SRC may result in a range of clinical signs and symptoms that may or may not involve loss of consciousness [14]. Resolution of physical and mental symptoms typically follows a sequential course, with most adults recovering within 7–10 days [2,14,15]. However, some athletes show prolonged symptoms lasting for months or years and a subset never fully recover [16]. Concussed athletes who do not recover within a certain timeframe are said to suffer from postconcussion syndrome (PCS). While the definition of PCS is debated, three or more postconcussive symptoms lasting 3 months or longer is commonly used [17–19]. We used the ICD-10 criteria for PCS [19]. Previous concussions, a high number of initial symptoms and an ignorance toward return-to-play recommendations are linked to a longer duration of PCS [16,20,21]. The likelihood of developing chronic or even progressive symptoms has been emphasized by findings indicating an association between repeated SRCs and impaired long-term mental health in athletes [20–24].

In recent years, reports of Swedish ice hockey players who prematurely have had to end their career have increasingly appeared in the lay press [25]. To the best of our knowledge, there are no previous long-term evaluations of ice hockey players ending their career due to PCS and their overall well being has not been studied in detail. A recently accepted qualitative study showed that players who are forced to retire from ice hockey struggle with forming their identities and finding sources of meaning for their lives [26].

In the present study, we assessed the postconcussive symptom burden, quality of life and post-traumatic stress in former Swedish professional ice hockey players with prior concussions. The players all ended their careers following to a history of multiple concussions, either due to a fear of attaining additional concussions or to a high symptom burden that prevented further play. We hypothesized that the retired players suffer from persistent symptoms and that outcomes in terms of quality of life and post-traumatic stress is worse in players ending their careers due to a high symptom burden.

## Materials & methods

### Ethics

The study was conducted in accordance with the Declaration of Helsinki and approved by the Regional Ethics Committee of the University of Gothenburg, Gothenburg, Sweden (Dnr 646-14). All athletes signed a written consent prior to participation.

### Participants

We aimed to identify players who retired from Swedish ice hockey because of SRCs. Information about the study was sent to all ice hockey clubs in Sweden, the medical staff of the teams in the male and female elite leagues and all members of The Swedish Ice Hockey Medical Association. There are about 50,000 players listed in the approximately 400 ice hockey clubs in Sweden. We asked to identify ice hockey players who retired from the sport as a result of SRC. The identified players gave consent to being contacted by, or were encouraged to contact, one of the authors (Y Tegner) if they were interested in partaking in the study. In addition, all included players were asked if they were aware of other players who had had to end their ice hockey careers due to SRC. The internet was also searched for players who had stopped playing ice hockey due to SRC. If such players were identified, the ice hockey club where the player had last played at was contacted and recruitment was made using the same method as for the other participants. The study design was explained to the participants over the telephone and via a written explanation. Players who gave written consent to participate received the four questionnaires described below. Athletes were recruited between 2014 and 2019.

### Outcome measures

A questionnaire addressing the athletic career and concussions sustained during sports was used. The following questions were included: sex, age, level of play, number of years playing, reason for terminating the sporting career (symptoms or a concern about sustaining additional concussions), number of concussions, age at first concussion, age at last concussion, age at retirement, time from last concussion until termination of the sporting career, it they were more susceptive to concussions, hospitalization due to concussion and current work situation (full-time or no work). Level of play was recorded as professional, semiprofessional or amateur. Professional players were defined as having competed full-time without any other work in the highest leagues in Sweden or in a corresponding international league. Semiprofessional players competed and played part-time in a lower league in Sweden. Amateurs work or study full time and play in a lower league in Sweden, without being payed.

This study includes athletes who retired from play due to concussions, either due to a high burden of symptoms that made it impossible to continue to play, or due to concerns of sustaining additional concussions. This is the main separating factor in the study; henceforth, the groups will be referred to as group Concussion Symptoms (gCS) and group Concussion Concerns (gCC).

#### The Sport Concussion Assessment Tool graded symptom checklist

This graded symptom checklist has been a part of the Sport Concussion Assessment Tool (SCAT) since its first version [27–30]. This symptom checklist is self-administered. It evaluates 22 different symptoms in seven rankings on a Likert scale, where 0 is unaffected and 6 is affected to the highest degree by the symptom. The scores are summarized as number of symptoms (maximum 22) and symptom severity (maximum 132).

#### The Short Form Health Survey quality of life

The Short Form Health Survey (SF-36) has 36 questions measuring different health domains, low scores imply a poorer health-related quality of life. Scores are recorded between 0 and 100. The SF-36 is suitable for self-administration and can be completed in 5–10 min. For comparison, there are sex- and age-normative control groups [31]. The Swedish version of the instrument has been validated [31] and SF-36 has previously been used in the evaluation of mTBI [32,33]. The SF-36 uses eight subscales with four scales relating to functional outcomes, three scales relating to well-being and one overall health scale. Functional scales include: physical functional, indicating limitations in physical activity; role physical, indicating problems with work and daily activities due to physical health; social functioning, indicating interference with normal social activities due to physical or emotional issues; and role-emotional, indicating problems with work or daily activities due to emotional problems. Scales relating to well-being include: bodily pain, indicating limiting pain; vitality, indicating fatigue; and mental health, indicating feelings of nervousness or depression. The overall measure of health is the scale of general health [34].

#### The impact of event scale-revised post-traumatic stress

The impact of event scale-revised (IES-R) was developed for self-reporting of post-traumatic stress disorder [35,36] and has now been validated for several different types of physically and psychologically traumatic events. It is widely used to measure subjective distress in relation to a traumatic event [37]. The Swedish version has been implemented and validated for mTBI, whiplash injury and other traumatic events [38–42]. We classified a traumatic event as the realization that they had to give up ice hockey pre-maturely due to the effects of SRCs.

The scale consists of 22 questions in five different scores; 0 is unaffected and 4 is extremely distressed. The scores are separated into three subscales: intrusion (eight items), avoidance (eight items) and hyperarousal (six items). The maximum mean score on each of the three subscales is 4, the highest total mean IES score is 12 and the highest total sum is 88. Total scores of 24–32 are considered subclinical [43] and scores >32 considered clinical [44] for post-traumatic stress disorder.

### Statistics

Data were analyzed for normal or skewed distribution using Shapiro–Wilk test. Normally distributed data were compared using means and *t*-tests and skewed data with Mann–Whitney U test for pairwise comparisons. Categorical and binominal values were assessed with chi-squared tests. To determine the associations between SCAT5 and SF-36 and IES-R, respectively, simple linear regression was used and Pearson’s correlation coefficients calculated.

Probability values ≤0.05 were considered statistically significant. All statistical analysis was conducted in SPSS (SPSS Inc., version 25, IBM, NY, USA).

## Results

### Demographics & clinical characteristics of study participants

95 players were identified and verified to have retired from ice hockey due to concussions. Of these 82 responded, of whom five decided not to partake and one player could not define the reason for retirement and was therefore excluded. Of the 76 participating athletes, 30 answered the questionnaires in written form and 46 digitally on the internet.

**Figure 1.**
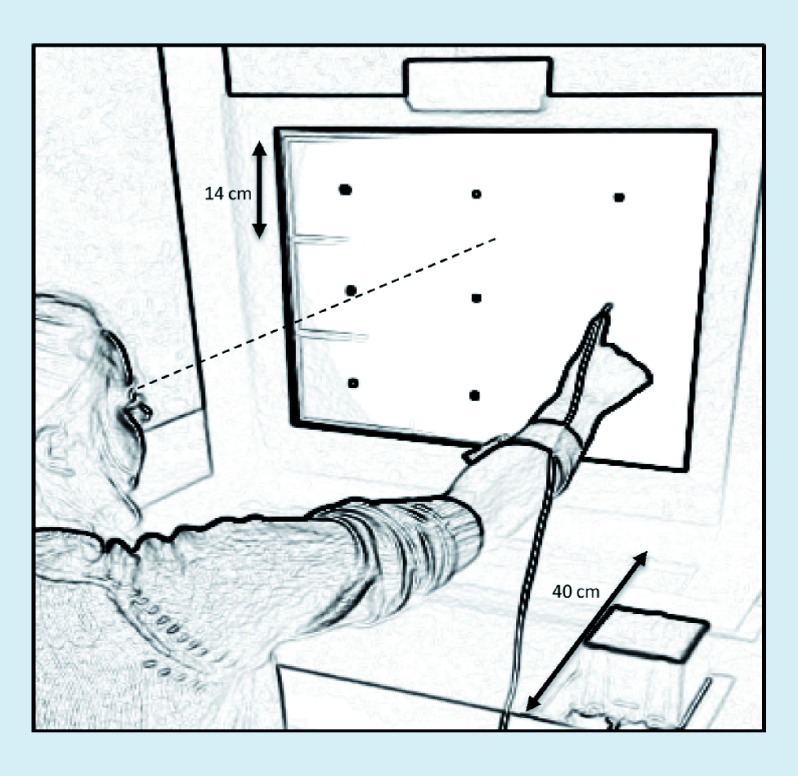
Concussions. gCC: Group Concussion concerns; gCS: Group Concussion Symptoms.

A total of 70 (92%) were male and six (8%) were female. The players retired on average 5 years (range: 1 to 28 years) prior to the present investigation. The age ranged from 19–56 years (mean 30 ± 8 years). The athletes had predominantly played at a professional (41%) or semiprofessional (55%) level, while only three athletes (4%) played at an amateur level. Duration of the career was 19 ± 6 (range: 5 to 30) years. Age at first concussion was 16 ± 5 (range: 5 to 31) years and age at last concussion was 25 ± 6 years. The players sustained 6 ± 3 (range: 1 to 20) concussions during their careers (Figure 1). Most athletes (82%) subjectively experienced an increased susceptibility to attain an additional concussion injury following each SRC. A total of 49 (64%) of the athletes had been admitted to hospital due to concussion.

The predominant reason for terminating the career was persistent postconcussive symptoms (gCS: 58%). The remaining athletes (gCC: 42%) stated that the reason for termination was a concern of attaining additional concussions. It took 6 ± 5 months from the last concussion until the decision to terminate the career was made. The mean age at retirement was 25 years (range: 13 to 39). After retirement from the sporting career, 61% worked full-time in another job.

In terms of demographics, there were some differences between gCS and gCC, namely the ability to work full time and whether they had been hospitalized due to concussion (Table 1).

**Table 1.** Demographics.

	gCS n = 44	gCC n = 32	p-value
Age	30 (7.8)	29 (7.3)	0.597
Years of play	19 (6.2)	18.5 (5.3)	0.538
Number of concussions	5.7 (3.0)	6.3 (3.2)	0.382
Age at first concussion	16 (4.5)	15 (4.4)	0.342
Age at last concussion	25 (7.1)	25 (5.4)	0.847
Months from last concussion to retirement	5 (2–8)	4 (1–6)	0.293
Sex, male	39	31	0.399
Level of play – Professional – semiprofessional – amateur	41% 57% 2%	41% 53% 6%	0.809
More susceptive to concussions, yes	77%	88%	0.466
Hospitalization due to concussion, yes	77%	47%	0.021
Work full-time, yes	41%	88%	<0.001

Demographics of participants in the study, separated into gCS and gCC. Presented as mean and standard deviations or median and interquartile range. Distribution is presented in percent. Distribution between groups are similar, except concerning hospitalization due to concussion and working full-time.

gCC: Group Concussion Concerns; gCS: Group Concussion Symptoms.

### Retired ice hockey players have persistent postconcussive symptoms

**Figure 2.**
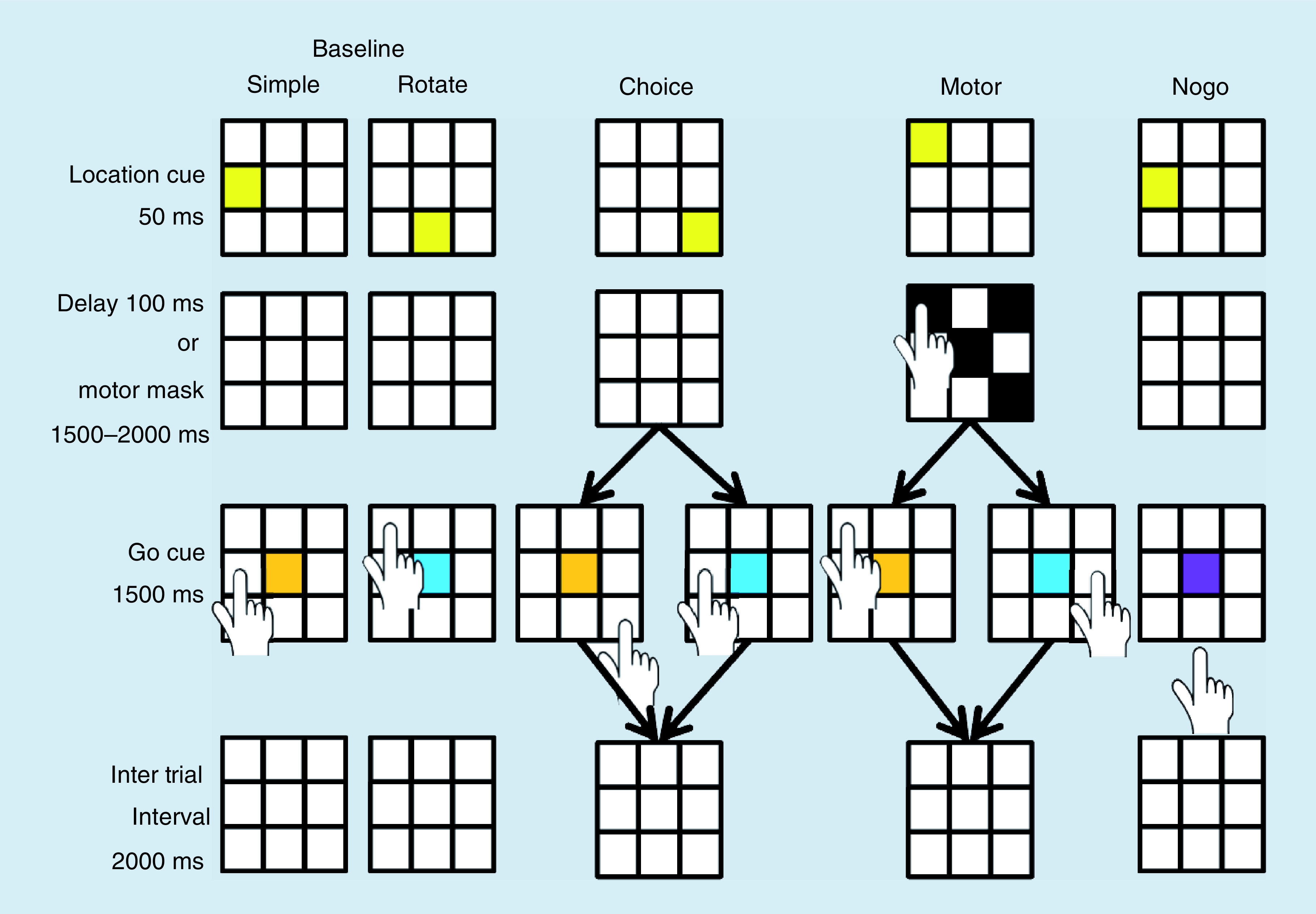
The Sport Concussion Assessment Tool for grading of symptoms. Symptom severity is reported in seven scales, 0 is no symptom, 1 represents a mild degree of suffering from the symptom and 6 the maximum suffering from the symptom. **(A)** Shows the symptoms in group Concussion Symptoms and **(B)** shows the symptoms in group Concussion Concerns.

All participating athletes (n = 76) completed the graded symptom checklist. The symptoms and their severity are listed in declining incidence (Figure 2A &B), with 86% of subjects suffering from nervousness or anxiousness as the most frequent symptom. The median number of symptoms was 16 (interquartile range; IQR: 11–21) and all but three athletes suffered from any symptoms. Symptom severity scores ranged from 0 to 102, with a median of 39 (IQR: 15.5–69). Age and number of SRCs did not correlate with symptom severity score: p = 0.416 and p = 0.474, respectively.

In gCS, the median number of symptoms was 20 (IQR: 14–21) and the median symptom severity score was 65 (IQR: 36–80). In gCC, the median number of symptoms was 11 (IQR: 6–17) and median symptom severity was 26 (IQR: 7–39), both significantly lower when compared with gCS (p < 0.001 for both analyses).

### Ice hockey players have reduced quality of life following SRC

**Figure 3.**
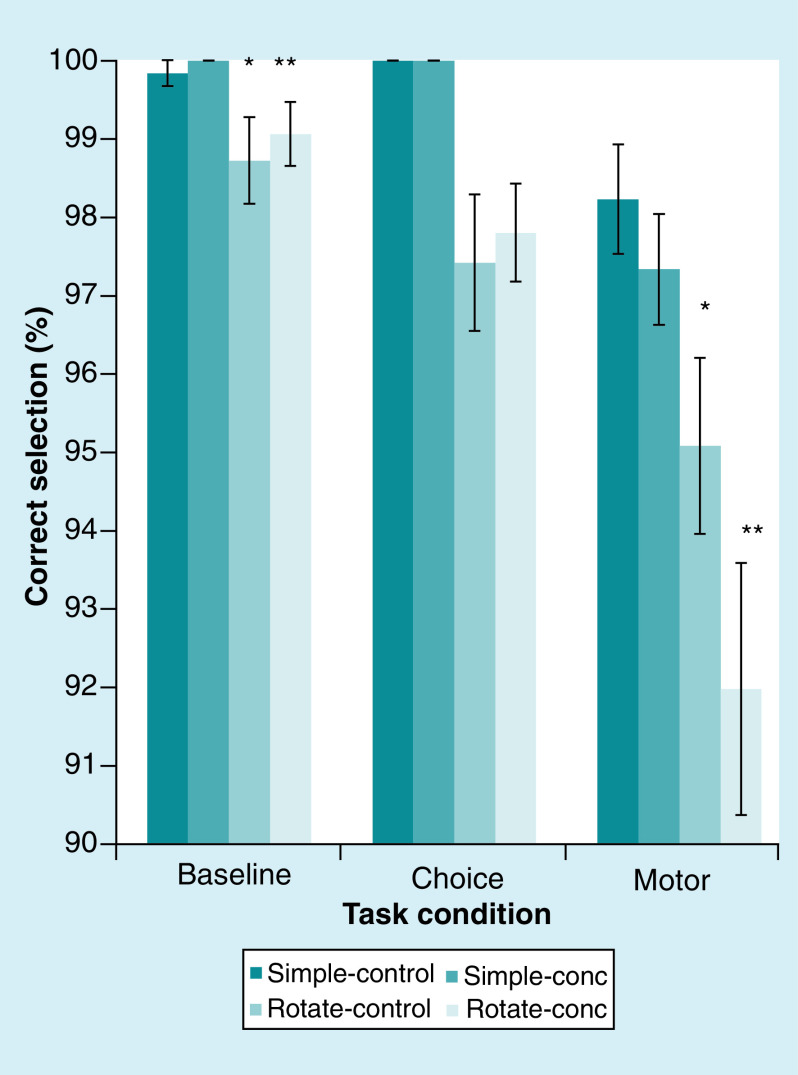
The Short Form Health Survey assessing health-related quality of life. Eight subscales; PF, RP, SF, RE, BP, VT, MH and GH. Scores are recorded between 0 and 100, higher scores indicating better health-related quality of life. The scales are presented with means and a 95% confidence interval in age- and gender-matched norms, gCC and gCS. In **(A)** males aged 15–44 are shown. There are 38 males in gCS aged 15–44 and 30 males in gCC aged 15–44. Significant differences are seen when comparing gCS to gCC, gCS to norms and gCC to norms. In **(B)** females aged 15–44 are shown. There are five females in gCS aged 15–44 and one female in gCC aged 15–44, therefore, a large confidence interval can be seen in gCS and it is not calculated for gCC. BP: Bodily pain; gCC: Group Concussion Concerns; gCS: Group Concussion Symptoms; GH: General health; MH: Mental health; PF: Physical function; RE: Role emotional; RP: Role physical; SF: Social function; VT: Vitality.

As SF-36 is dependent on sex and age, we evaluated males and females separately in age groups and gCS with gCC. In comparison with norms [31], males aged 15–44 in gCS differed significantly in all domains of quality of life and males aged 15–44 in gCC differed significantly in role physical, general health, vitality, social functioning, role-emotional and mental health. Women aged 15–44 in gCS differed significantly in role physical, bodily pain, vitality, social functioning and role-emotional. Males aged 15–44 differed significantly between gCS and gCC in all domains but role-emotional and mental health. Women in gCC and males aged 45–64 were too few for statistical analyzes (Figure 3A & B). Comparing all male and female players, separated into gCS and gCC, the groups significantly differed in all domains but role-emotional (Table 2); however, role-emotional differed significantly from norms in both gCS and gCC.

**Table 2.** Short Form Health Survey.

	gCS mean (95% CI)	gCC mean (95% CI)	p-value
Physical functional	84 (79–88)	96 (94–98)	<0.001
Role physical	35 (23–48)	73 (62–84)	<0.001
Social functioning	59 (51–66)	80 (73–86)	<0.001
Role emotional	59 (47–72)	71 (57–85)	0.23
Bodily pain	51 (43–58)	78 (71–86)	<0.001
Vitality	40 (33–47)	63 (57–69)	<0.001
Mental health	63 (57–69)	74 (69–79)	0.01
General health	55 (48–62)	72 (66–78)	0.001

The Short Form Health Survey-36 assessing health-related quality of life. Scores are recorded between 0 and 100, higher scores indicating better health-related quality of life.

The scales are presented with means and a 95% CI. All scales but role emotional differ significantly between gCS and gCC.

gCC: Group Concussion Concerns; gCS: Group Concussion Symptoms.

### The impact of event scale-revised

Out of the 76 included athletes, 68 men and six women completed the IES-R form. Questions and the differences between gCS and gCC are listed in Table 3. There was no statistical difference between men and women concerning total IES-R scores; men had a mean score of 27.7 and women 27.5 (p = 0.987). When comparing gCS with gCC, there were several differences, including a higher total score in gCS compared with gCC (31.7 vs 22.2; p = 0.028) and a higher mean hyperarousal score in gCS (1.4 vs 0.7; p = 0.001). There were no statistically significant differences between gCS and gCC in mean intrusion (1.5 vs 1.2; p = 0.171) or avoidance (1.4 vs 1.1; p = 0.102) scores.

**Table 3.** The impact of event scale-revised.

Items	gCS, n = 43		gCC, *n = 31*		p-value
	Total	Mean	Total	Mean	
Intrusion	12.1	1.5	9.6	1.2	0.171
Avoidance	11.2	1.4	8.5	1.1	0.102
Hyperarousal	8.4	1.4	4.1	0.7	0.001
Total score	31.7	1.4	22.2	1.0	0.028

The IES-R. Three subscales; intrusion, avoidance and hyperarousal.

Scale-scores can range from 0 to 4 with a maximum total score of 88. Items are presented as a mean value for each item, subscale and total. High values mean a high burden of post-traumatic stress. The athletes are separated into gCS and gCC. There is a significant difference between gCS and gCC in subscale hyperarousal (p = 0.001) and in the total score (p = 0.028).

Two former players did not answer this questionnaire.

gCC: Group Concussion Concerns; gCS: Group Concussion Symptoms; IES-R: Impact of event scale-revised.

### Persistent postconcussive symptoms correlate with reduced quality of life

Correlations between SCAT symptom severity and number of symptoms with total scores of SF-36 and IES-R, was calculated using Pearson’s correlation coefficient. Significant correlations between symptom severity and SF-36 (r = -0.80, CI: -1.30 to -0.91; p < 0.001), number of symptoms and SF-36 (r = -0.78, CI: -0.28 to -0.19; p < 0.001), symptom severity and IES-R (r = 0.54, CI: 0.54–1.16; p < 0.001) and number of symptoms and IES-R (r = 0.51, CI: 0.10–0.24; p < 0.001) were observed. We also corrected for age and time since last concussion and found each correlation to be significant and even stronger: symptom severity and SF-36 (r = -0.82, CI: -1.33 to -0.93; p < 0.001), number of symptoms and SF-36 (r = -0.79, CI: -0.29 to -0.20; p < 0.001), symptom severity and IES-R (r = 0.61, CI: 0.62–1.30, p < 0.001) and number of symptoms and IES-R (r = 0.58, CI: 0.12–0.27; p < 0.001). Hence, age and time since last concussion does influence these outcome measures.

**Figure 4.**
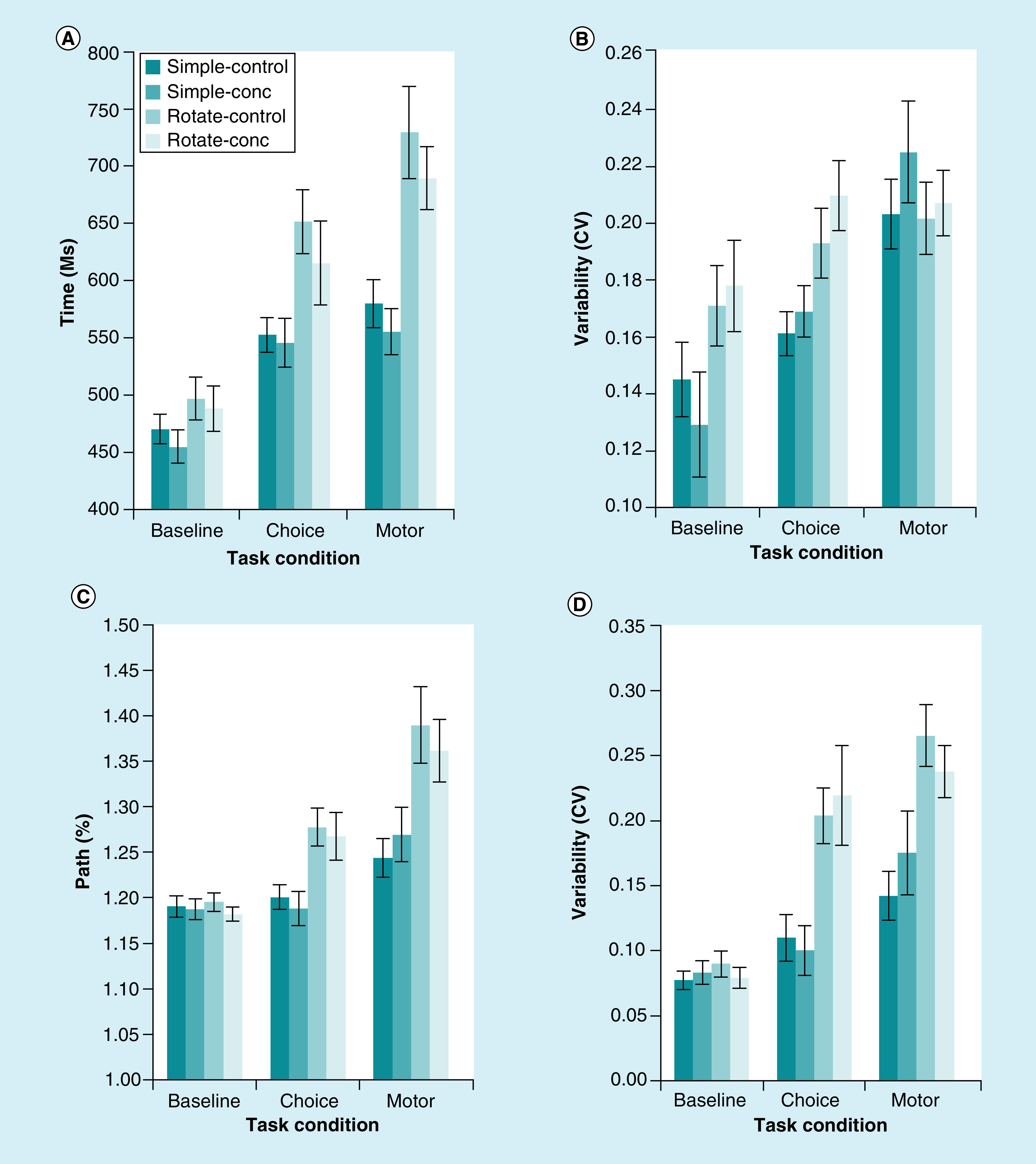
Correlation Sports Concussion Assessment Tool with Short Form Health Survey and impact of event scale-revised. Correlation between the SCAT with the SF-36 and the IES-R in gCS and gCC. **(A)** Higher values on SCAT symptom severity correlates negatively with lower values on SF-36. High values on SCAT indicates a high symptom burden and low values on SF-36 indicates a bad rating of health-related quality of life. The correlation is significant for gCS (p < 0.001), for gCC (p < 0.001) and hence for all athletes (p < 0.001). **(B)** Higher values on SCAT symptom severity correlates positively with higher values on IES-R. High values on IES-R indicates a high burden of post-traumatic stress. The correlation is significant for gCS (p < 0.001), although not for gCC (p = 0.20). Calculated for all athletes there is a significant correlation (p < 0.001). **(C)** Higher values on SCAT numbers of symptoms correlates negatively with lower values on SF-36. High values on SCAT indicates more symptoms and low values on SF-36 indicates a bad rating of health-related quality of life. The correlation is significant for gCS (p < 0.001), for gCC (p < 0.001) and hence for all athletes (p < 0.001). **(D)** Higher values on SCAT numbers of symptoms correlates positively with higher values on IES-R. High values on IES-R indicates a high burden of post-traumatic stress. The correlation is significant for gCS (p < 0.001) and almost for gCC (p = 0.053). Calculated for all athletes there is a significant correlation (p < 0.001). gCC: Group Concussion Concerns; gCS: Group Concussion Symptoms; IES-R: Impact of event scale-revised; SCAT: Sports Concussion Assessment Tool; SF-36: Short Form Health Survey.

Analyzed separately, gCS had significant correlations between symptom severity and SF-36 (r = -0.77, CI: -1.31 to -0.77; p < 0.001, Figure 4A), number of symptoms and SF-36 (r = -0.77, CI: -0.25 to -0.15; p < 0.001, Figure 4C), symptom severity and IES-R (r = 0.62, CI: 0.54–1.26; p < 0.001, Figure 4B) and number of symptoms and IES-R (r = 0.55, CI: 0.08–0.22, p < 0.001, Figure 4D). In gCC, significant correlations were found between symptom severity and SF-36 (r = -0.66, CI: -1.27 to -0.51; p < 0.001, Figure 4A) and number of symptoms and SF-36 (r = -0.69, CI: -0.39 to -0.17; p < 0.001, Figure 4C); however, not between symptom severity and IES-R (r = 0.24, CI: -0.17–0.77; p = 0.20, Figure 4B) or number of symptoms and IES-R (r = 0.35, CI: -0.002–0.27; p = 0.053, Figure 4D).

Number of concussions did not correlate with symptom severity (p = 0.474), number of symptoms (p = 0.570) or SF-36 (p = 0.607). However, number of concussions correlated positively with IES-R (r = 0.23, CI: 0.001–0.077; p = 0.047).

## Discussion

In this study, we evaluated ice hockey players who retired from the game due to multiple concussions, either because of concerns over sustaining additional concussions or due to a high symptom burden. Most athletes had been playing at a high level for many years and had attained several concussions. The main findings were that previously concussed former ice hockey players are suffering from a substantial symptom burden and that the symptoms are dependent upon the reason for the termination of their career. Ice hockey players retiring from their career due to persistent postconcussion symptoms had a substantially lower quality of life compared with population-based data, while less symptomatic players retiring due to concerns of potential future concussions did not. The evaluation of the athletes in this study was performed a mean of 5 years after their careers had been terminated and imply that concussion-related problems persist a long time after injury which is in line with previous results [16]. These results emphasize that repeated concussions, when producing many and severe persisting symptoms, have a substantial prolonged effect on a player’s well-being and ability to work.

More symptoms and a higher symptom severity were reported by gCS compared with gCC. This was expected, since gCS retired due to a high symptom burden. Nevertheless, it validates that athletes are consistent in the evaluation of their health.

The IES-R results may indicate that there is a significant difference in the post-traumatic stress response between the two groups. However, when analyzing the results in detail, this may not be the final conclusion. When the IES-R results were analyzed separately, there were nonsignificant results on two of the subscales and the third (hyperarousal) was markedly different, resulting in a significant difference when all scales were combined. It is thus possible to infer that there may be less problems with mood and vigilance in the gCC compared with the gCS. Neither of the groups reported symptoms of post-traumatic stress. Measured with SF-36, a trend of low quality of life in both groups was observed compared with norms, gCS worse than gCC. Similar results of reduced quality of life estimated with SF-36 have been previously observed in mTBI patients [33]. The worst scores are in the domains of role physical and vitality, indicating difficulties with work or daily activities due to poor physical health and feelings of tiredness [34]. The athletes have impaired work capacity compared with the general population in Sweden (2019: 3% full-time sick-leave, 6.8% unemployed) according to the National Insurance Office [45] and the Central Bureau of Statistics [46]. In gCC, 88% had a full-time job compared with just 41% of gCS. Among the most frequently reported symptoms were mood disorders, pain, fatigue and difficulty concentrating. These symptoms are often exacerbated by physical and mental activity, a noisy environment or social cohesion – situations that are impossible to avoid in most occupations. The incapability of maintaining a job can potentially have a negative impact on perceived quality of life, amplifying the effect caused by a high burden of symptoms. In Sweden, the majority of players continue working in other professions at least in part since the salaries are not sufficient for life-long financial support. Thus, it is unlikely that there are financial causes for the lack of employment.

A study of athletes with SRC found that higher rating of the fear of re-injury correlated with higher total and affective postconcussive symptoms [47]. This indicates that athletes with a greater concern of additional SRC may rate their symptoms as more severe, which may influence results in both gCS and gCC but particularly in gCC. It has previously been suggested that females are particularly sensitive to concussions, generally reporting more symptoms and having longer recovery times [48]. However, we were only able to include six female players and therefore could not analyze any potential difference between sexes. This was likely due to there being fewer female elite ice hockey players than males. A nonanalyzed factor in this study, previously shown to influence the recovery and lead to higher rating of symptoms, is psychiatric disease [49,50]. Depression, anxiety and attention disorders are quite common and can have a great impact on similar outcome measures, therefore psychiatric disease may be included as demographics in future studies. There are diverging results concerning age; according to a systematic review some studies indicate that young age is related with worse outcome, although some report no differences [50]. In this study, we did not observe any relation between age and a higher symptom burden.

This study implicates an association between concussion and long-term outcomes and that athletes with a high symptom load are at the greatest risk. No association between number of concussion and symptom burden was observed; however, we have no information about subconcussive blows, which may potentially affect symptom burden. In a previous study of football players with and without concussions and healthy nonfootball controls, hippocampal volumes were compared. A dose-related effect was seen, with concussed football players having the smallest volumes, followed by nonconcussed football players and then controls [51]. Similar results indicating a dose-related effect has been previously shown with regards to many aspects of concussion and subconcussion pathology, such as white matter integrity [52], vestibulo-ocular function [53] and performance on neuropsychological tests [54]. Alas, athletes tend to underreport their concussion symptoms and continue playing at the time of the event [55,56], sometimes due to expectations of others [57]. Thus, in order to improve awareness, education targeting players, coaches, parents and the public is desirable. Other suggested approaches to reduce the incidence of concussion in ice hockey are rule changes, such as a penalty for checking to the head and stricter enforcement of existing rules [58]. The results of our study suggest that the number and the severity of symptoms may help to guide the management of athletes with concussions. We cannot identify any definitive thresholds concerning the numbers of symptoms or their severity. A recently published study shows that increasing symptom severity leads to longer return to play when symptom severity is >10 on SCAT3 [49], which may act as a guiding cut off value for a more substantial rehabilitation protocol.

It is of the greatest importance to identify athletes at increased risk of SRC to pause competition play and to engage the player in a rehabilitation protocol aiming for safe return-to play.

## Limitations

This study has several limitations. It has earlier been indicated that athletes are reluctant to report concussions and concussion symptoms, hence the rate of injuries can be underestimated [55]. In this study, we did not have access to past medical records providing information on previous or current management and rehabilitation, or other subjective measurements of concussions and symptoms. The status of those who chose not to partake is unknown. Plausibly, athletes with a higher symptom load seek more help for their difficulties and are more likely to participate in evaluations. We could have missed concussed athletes, due to underreporting or missing data on the history of concussion. Young athletes playing in a lower league with one or few concussions might be especially prone to end play without reporting the reasons why. These are all potential selection and recruitment biases.

While it is possible that participants did not follow the intended guidelines for answering the questionnaires, both groups had the same conditions when participating.

The questionnaires used in this study have all been shown good validity and reliability [28,29,31–37,43]. The IES-R was constructed to evaluate a specific injury. We used it in a different way, as the players were told to consider the traumatic event to be the fact that they had to retire from ice hockey. The validity of this use of the IES-R can be criticized, although it is plausible that it provides view of the anxiety and stress levels in former ice hockey elite players.

We were limited by the available normative data since we did not collect data on controls without concussions. SF-36 has a large sample of age and gender matched norms; however, for IES-R, suitable normative data could not be obtained. Further, norms are matched to the players in consideration to age and gender, but probably differ substantially concerning sports history and may therefore not be reliable for comparison.

## Future perspective

SRC is an increasingly recognized issue with growing concerns of long-term consequences, such as persisting symptoms and a lower quality of life. Ice hockey is a sport played at high speed with forceful body-to-body contact, which puts the athlete at risk of high-energy impacts. Injuries are common and the incidence of SRCs is increasing. In this study, the long-term consequences of SRC included prolonged symptoms and a lower quality of life. The number of and the severity of symptoms may act as a guide when the player should or should not return to play. This may seem intuitive, yet specific cut-off values are desirable to facilitate the management and hence further studies are warranted. Other factors, such as biomarkers, neurocognitive assessment, refined neuroimaging and anti-inflammatory drugs, need to be further studied to evaluate if these can predict or aid recovery.

## Conclusion

We conclude that athletes with SRC and postconcussive symptoms experience long-term physical and mental difficulties that affect their quality of life. The athletes were affected by concussions and to some extent had unfavorable sequelae, athletes with a higher symptom burden had worse outcomes. These data indicate that it is important to continuously evaluate symptom burden to guide this decision and that it may be favorable to retire earlier when the symptom burden is reduced, to avoid developing persistent postconcussion symptoms leading to a lower quality of life.

**Summary points**

**Participants**

- A total of 76 ice hockey players who retired due to sports-related concussion (SRC) were included.
- The reason for retirement was in 58% due to high symptom burden group Concussion Symptoms (gCS) and in 42% due to concern of sustaining additional concussions group Concussion Concerns (gCC).
- After retirement 61% worked full time in another job.

**Symptom evaluation**

- 73 out of 76 players had symptoms.
- There was more symptoms and worse graded symptoms in gCS compared with gCC.

**The Short Form Health Survey**

- Players rated a worse quality of life when compared with norms.
- gCS rated a worse quality of life when compared with gCC.

**The impact of event scale-revised**

- gCS had a higher hyperarousal and total score when compared with gCC.
- The total score of gCS is considered subclinical for post-traumatic stress disorder.

**Conclusion**

- Athletes retiring due to SRCs suffer from long-term symptoms and a lower quality of life.
- Retired athletes with a higher symptom burden had worse outcomes.
- High symptom scores correlated with lower quality of life and higher rated post-traumatic stress.
- We recommend that symptom burden should be continuously evaluated and guide the decision to retire from play or not.
